# Coprolite Multimodal Analysis: A Tool for *Hyaenid* Predator Identification

**DOI:** 10.3390/ani15081145

**Published:** 2025-04-16

**Authors:** Yannicke Dauphin, Jean-Philip Brugal

**Affiliations:** 1 IRN 0871 TaphEN, CNRS-INEE, 13097 Aix-en-Provence, France; jean-philippe.brugal@univ-amu.fr; 2 Institut de Systématique, Evolution, Biodiversité (ISYEB), UMR 7205 CNRS, Sorbonne Université, EPHE, Muséum National d’Histoire Naturelle, 75005 Paris, France; 3 Department of Biomaterials, Max-Planck Institute of Colloids and Interfaces, 14476 Potsdam, Germany; 4 UMR 7269 LAMPEA, CNRS, Aix Marseille Univ., Minist. de la Culture, 13097 Aix-en-Provence, France

**Keywords:** coprolite, modern, *Hyaenid*, FTIR, SEM, alteration

## Abstract

Paleontologists and archeologists reconstruct ancient ecosystems using data from carnivores’ food remains. Carnivores have evolved to employ two primary feeding strategies: consuming mostly meat and focusing on both meat and bones, and these strategies result in the production of different feces. Hyenas are exemplary meat-eaters and bone-crushers. While fecal characteristics like shape, color, size, and inclusions are often used for species identification, the detailed composition of hyena feces remains largely unexplored. To address this, we conducted a multimodal analysis of feces-like coprolites from four modern Hyaenid species, using scanning electron microscopy (SEM) and Fourier transform infrared spectrometry (FTIR). This approach allowed for the detection and quantification of the proportions of calcium phosphate/carbonate, silts, organic matter, and crystallinity in the coprolites. Our preliminary findings suggest that multivariate statistical analysis of these components could provide a reliable method for species identification based solely on fecal content, results which can be applied in research on fossil materials.

## Simple Summary

Coprolites are mineralized feces which can be recorded in the fossil record, coming sometimes from herbivores, but mostly from carnivores who eat the meat and bones of their prey. They can provide important information for archeologists and paleontologists about past ecosystems. Coprolites are well preserved in relation to the calcium and phosphate composition of bones. It is essential to better characterize the feces of modern carnivores for referential purposes, and here, we specifically focus on the members of the *Hyenidae* family, who are some of the more efficient bone-eaters among predators (the family comprises three carnivorous species, and one that is strictly insectivorous). Several complementary methods are used (SEM, FTIR spectrometry, X-ray spectroscopy) in combination with multivariate statistical analysis. Our results describe the chemical and morphological features of the feces of these four species, and provide comprehensive data that allow for the identification of the species, which could be used as a reference for application in research on fossil materials.

## 1. Introduction

Reconstructions of paleoenvironments typically involve morphological and compositional analysis of terrestrial and aquatic sediments and biological materials. Most studies are dedicated to paleoclimates and reconstructions of paleoflora and paleofauna; the ancient behaviors and inter-relationships of plants and animals are also informative. For a long time, comparative anatomy of skeletal remains, mainly skulls and teeth, was an essential method to discriminate herbivorous and carnivorous animals. The structure and composition of bone do not differ strongly among vertebrates. Nevertheless, minor variations in elemental chemistry and isotopes are tied to diet [1,2]. Unfortunately, the composition and structure of bone vary throughout the lifespan of an animal (remodeling), meaning that such analyses reflect only a snapshot, rather than providing a comprehensive view of the growth of the organism. Moreover, taphonomical and diagenetical processes also tend to modify the bone composition in the fossil record.

Analyzing the droppings, or feces, of rare and elusive animals can provide valuable insights into their diet and health. Fossilized feces, known as coprolites, offer snapshot data on both the producer and its food sources [3]. They concern a wide diversity of invertebrates and vertebrates (fish, reptiles, birds, mammals), and they are common in the Pleistocene record, especially from hyena representatives. Several categories of criteria can be used to identify the producer: size [4], morphology, inner structure [5,6,7], content (identification of fragments), and composition [8,9,10,11,12,13,14]. Pollen, hairs, seeds, parasites, and phytoliths are recovered from coprolites [15,16], and even when they have been accidentally ingested, they reflect the environment in which the producer lived. It is important to note that most studies typically rely on a combination of these criteria, rather than just a single one, to improve the accuracy of fecal producer identification.

X-ray diffraction, infrared spectrometry, energy-dispersive spectrometry, and molecular analyses (lipids, proteins, DNA) have been used on a small number of samples. At the same time, attempts to identify the taxonomic origin of coprolites have yielded questionable results [17,18,19,20].

This study diverges from the typical analysis of feces or coprolites, shifting the focus from reconstructing the producer’s diet and environment to identifying the animal that produced the feces. Notably, modern feces from carnivores that ingest bones (including those examined in this study) are already transformed—they are often solid, stone-like, and contain hardened contents. Under favorable conditions, such as in rock shelters, dens, caves, or burrows, these feces can be well preserved and classified as coprolites. Hyenas serve as a well-documented example of carnivores with readily available coprolites. The family *Hyaenidae* includes four extant species: three carnivorous species and one nocturnal insectivorous species. Given the abundance of modern hyena coprolites, we pose a key question: can we distinguish the droppings of closely related hyena species based on their morphology, content, and bulk composition?

## 2. Materials and Methods

The Hyenidae family comprises four genera and four species: *Hyaena hyaena* (striped hyena), *Parahyaena brunnea* (brown hyena), *Crocuta crocuta* (spotted hyena), and *Proteles cristatus* (aardwolf) [21]. The first three species are carnivorous, but if necessary, *Parahyaena brunnea* is also able to eat insects, eggs, and wild fruits. *Proteles* does not scavenge or kill. It is a nocturnal insectivore (eating mainly termites). The genera *Crocuta* and *Hyaena* are well recognized in European Middle and Late Pleistocene records (ca. 780–10 ky), to which we can add *Pachycrocuta*, *Pliocrocuta*, and *Chasmaporthetes* during the Lower Pleistocene (ca. 2.6 My to 780 ky).

### 2.1. Materials

#### 2.1.1. Feces

**Figure 1:**
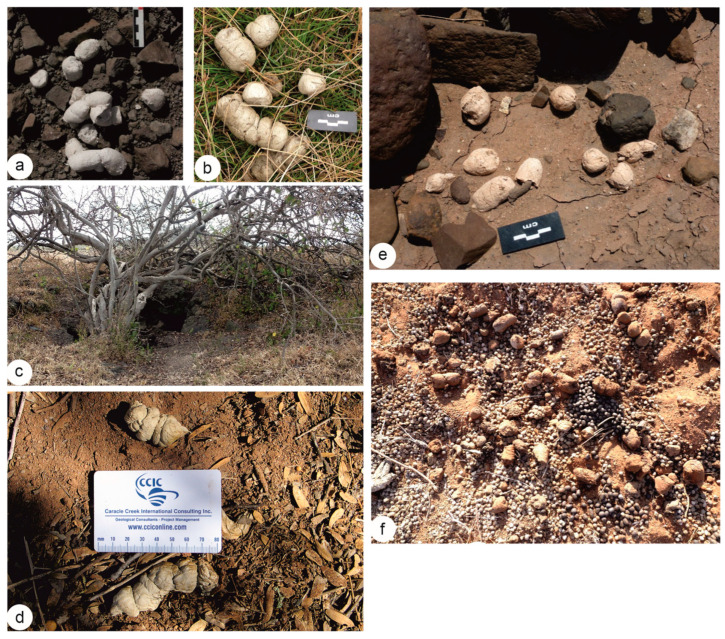
(**a**,**b**) *Crocuta crocuta*: (**a**) isolated drops from Kenya (Green Crater Lake), © JPB; (**b**) isolated drops from Djibouti, courtesy of J.B Fourvel. (**c**) *Crocuta crocuta*: latrine drops, close to caves in Soysambu (Kenya) © JPB. (**d**) *Parahyaena brunnea* drops from South Africa; image courtesy of G. Avery. (**e**) *Hyaena hyaena*: isolated drops from West Turkana (Kenya) HM-LA2C, © JPB. (**f**) *Proteles cristatus*: drops mixed with other ungulate feces in a latrine in Samburu (Kenya), © JPB.

The feces studied come from four extant hyena species, all originating from African countries. Most specimens were found in isolation, representing single defecation events (approximately ten segmented fecal pieces). Some samples were located near resting sites, such as underground dens (Figure 1).

Nine samples were from spotted hyena (*Crocuta crocuta*), primarily collected in Kenya, with additional samples from Tanzania, Chad, and Djibouti. Four samples from striped hyena (*Hyaena hyaena*) originated in Kenya, while three brown hyena (*Parahyaena brunnea*) samples came from South Africa and Botswana. Finally, two samples from aardwolves (*Proteles cristatus)* were collected from a known latrine site in Samburu, South Kenya (Table 1).

The spotted hyena, *Crocuta crocuta*, is arguably the most well-known African carnivore. This heavily built species (54–81 kg) inhabits diverse environments across sub-Saharan Africa, functioning as both a top predator and an efficient, socially coordinated hunter. Spotted hyenas can form large groups (clans) of more than 50 individuals [22,23].

The striped hyena, *Hyaena hyaena*, is a medium-sized carnivore with a wide distribution across Africa (north of and including the Sahel)—particularly East and Northeast Africa and West Africa—the Middle East (including Arabia), the Caucasus, Turkey, Central Asia, and the Indian subcontinent. These hyenas weigh between 23 and 35 kg, and inhabit open or thornbush areas in arid-to-semi-arid environments. Striped hyenas are opportunistic scavengers, with a diet that includes a wide variety of vertebrates, invertebrates, and even vegetables [24,25]. They are predominantly solitary animals, but are occasionally found in pairs.

The brown hyena, *Parahyaena brunnea*, is endemic to southern Africa. This medium-sized, dog-like carnivore weighs between 28 and 50 kg, and inhabits deserts, semi-deserts, and open woodland savannas. Primarily scavengers (especially from lion kills), brown hyenas are found in small social groups, ranging from solitary individuals with cubs to small groups. Their diet consists of a wide variety of vertebrate carrion, including ostrich eggs, insects, and wild fruits [25,26,27].

All three hyena species possess powerful jaws and heavy conical premolars. These dental adaptations enable them to efficiently crush bones and extract marrow and other nutrients. Hyenas are even capable of swallowing bone fragments and teeth of their herbivore prey. This bone-crushing ability is a key dietary adaptation reflected in their socio-ecological traits [28,29,30].

The aardwolf, *Proteles cristatus*, is a distinct and unusual *hyenid* species. It differs from other hyenas in its smaller size (7.5–10 kg) and slender morphology. These adaptations, along with its reduced premolars, reflect its unique insectivorous diet, primarily consisting of termites and ants. The aardwolf occurs in southern Africa, as well as in eastern and northeastern regions, inhabiting diverse habitats, but is often found in open grassy plains [31,32].

#### 2.1.2. Standards

The main food of hyenids is meat and bone, so it was essential that both mineral and organic references be used for comparison.

Small pieces of an untreated diaphysis of a femur of a wild boar (*Sus scrofa*) were used as a reference for bone. Hairs of a red-haired domestic cat were used for keratin (protein). Commercial chitin is typically extracted from crab exoskeletons, and is α-chitin, whereas chitin from insect exoskeletons is β-chitin; therefore, we used the wings of a hornet as a reference. Commercial analytical-grade standards are cholesterol for lipids, and bovine serum albumin (BSA) and insoluble type I collagen for proteins.

### 2.2. Methods

#### 2.2.1. Fourier Transform Infrared Spectrometry (FTIR)

Infrared analyses were performed using an FTIR spectrometer (Thermo Nicolet 6700, Thermo Fisher Scientific, Waltham, MA, USA) equipped with a diamond ATR module (SMART endurance). After the background had been collected, the solid sample was placed onto the small crystal area. Analyses were conducted in a destructive way: a piece of the superficial region (5 mm deep) of a coprolite was taken, then roughly crushed. A small fragment was placed on the ATR module, crushed, and pressed against the diamond by an articulated arm. For every coprolite, spectra of four different fragments were analyzed and averaged. It must be noted that ATR can measure spectra without the need for any sample preparation, such as polished surfaces or uniform thickness. For each spectrum, 200 scans were accumulated in the wavenumber range of 4000–700 cm^−1^, with a spectral resolution of 4 cm^−1^. The gain was set to 4. The spectrum baseline was corrected using the Crystal Sleuth routine, and the wavenumbers were identified by Spectragryph—optical spectroscopy software v1.2.16 (F. Menges, available online at http://www.effemm2.de/spectragryph (accessed on 11 November 2024).

FTIR spectra of the calcite and aragonite groups are characterized by three major bands (Figure S1): ν3 at 1429 cm^−1^ and the ν2 doublet 877–848 cm^−1^ are attributed to the carbonate ion CO_3_^2−^; ν4 at 713 cm^−1^ is attributed to calcite; and ν3 at 1471 cm^−1^, and the two doublets ν2 at 858–844 cm^−1^ and ν4 at 713–700 cm^−1^, are attributed to aragonite. The bands in these doublets are of unequal intensities. The weak carbonate ν1 band is 1012 cm^−1^ for calcite and 1083 cm^−1^ for aragonite [33,34].

In bioapatites (bone, dentin, and enamel), organic and mineral components are visible (Figure S1). Among the mineral components, phosphate and carbonate exist: 878 cm^−1^ (ν2) and 1457 cm^−1^ (ν3) for CO_3_; 565–605 cm^−1^, 961 cm^−1^ (ν1), and 1020 and 1100 cm^−1^ (ν3) for PO_4_. According to Rey et al. [35] and Pleshko et al. [36], the frequency of the 1020 cm^−1^ band assigned to HPO_4_^2−^ and CO_2_ in non-stochiometric apatite increases with an increase in the length of the apatite *c-*axis. The 1238 cm^−1^ band is assigned to amide III, 1540–1560 cm^−1^ to amide II, and 1652–1660 cm^−1^ to amide I. Regarding organic compounds, the main peaks attributed to proteins are the so-called amide I band (~1650 cm^−1^), the amide II peak (~1550 cm^−1^), and the amide III band (1310–1240 cm^−1^).

Bands between 1230 and 1265 cm^−1^ are assigned to sulfate [37,38], with the strongest bands being between 1220 and 1240 cm^−1^. OH-stretching modes lie in the 3400–3750 cm^−1^ region.

Cholesterol, chitin, keratin, collagen, and BSA show strong amide A bands (Figure S1). Amide I and amide II bands are strong in collagen, BSA, keratin, and chitin spectra [39]. Bands between 800 and 1100 cm^−1^ are strong in cholesterol [40] (Figure S1), and weak or absent in BSA spectra. Bands due to C–H stretching vibrations (2800–3000 cm^−1^) are stronger than those of amide A, amide I, and amide II in cholesterol, and are usually said to be characteristic of lipids [41]. Similar bands are present in polysaccharides and proteins, but they are weaker than amide bands.

Infrared (IR) analysis produces spectra with characteristic regions of high intensity: “bands”. They vary in width, like the amide I band between 1600 and 1700 cm^−1^. This variability allows for the use specific portions of a band to identify specific features within samples. However, the sheer number of usable regions across different studies to characterize the same property can be overwhelming.

Therefore, we developed a three-step method to select the optimal regions from FTIR data, considering both mineral and organic bands, for more focused and effective analysis.

#### 2.2.2. Statistical Analyses: Methods

Our analysis began with understanding the overall composition of the samples. We achieved this by analyzing the entire range of infrared data (FTIR spectra) using linear regression. Next, we aimed to identify specific regions within the spectra that could distinguish between the samples based on both their mineral and organic components. We started by performing a principal component analysis (PCA), focusing on specific features linked to the minerals present in the coprolites. To better visualize the relationships between the samples, we repeated the PCA, excluding the *Sus* bone and organic standards. We then followed the same process for the organic components. Finally, by combining insights from both mineral and organic analyses, we selected three key mineral and three key organic bands that would allow us to effectively identify the predator species.

#### 2.2.3. Scanning Electron Microscopy (SEM)

A part of the roughly crushed surficial region selected for FTIR analyses was spread on double-sided adhesive carbon disks mounted on 9 mm specimen stubs by “pressing” the stub over the crushed sample.

Sample observations were conducted using both secondary (SE) and backscattered electron (BSE) modes. Uncoated and gold-coated samples were examined using an FEI FESEM Quanta F600 Philips, Amsterdam, Netherlands under low vacuum. Gold-coated samples were also observed using a Zeiss Gemini LEO 1550 (ZEISS: Oberkochen, Germany). Both SEM methods were conducted at the Max Planck Institute of Colloids and Interfaces, Potsdam.

The BSE mode involves high-energy electrons being reflected or backscattered out of the specimen. Since atoms with a high atomic number (Z) are more strongly scattered than light ones, they appear brighter. Therefore, such images contain compositional information. Mineralized remains such as bone and teeth contain Mg, P, and other elements with atomic numbers that are higher than those of organic components (mainly H, N, C, and O). As a consequence, zones rich in minerals are brighter than zones rich in organic matter.

#### 2.2.4. Energy-Dispersive Spectrometry (EDS)

Elemental chemical composition was analyzed using a FEG-FIB-SEM AURIGA 40 Zeiss, located at IPGP (Paris, France), and a Quattro S Environmental scanning electron microscope (ESEM), located at the Max Planck Institute of Colloids and Interfaces (Potsdam, Germany).

Powdered fragments were sprinkled onto carbon adhesive tape. Excess particles were removed using a hand fan. The samples were carbon-coated and analyzed at 15 keV under a low-vacuum mode. Given the heterogeneity of individual coprolites, only selective analyses were performed to refine the chemical data obtained from FTIR analysis.

## 3. Results

### 3.1. Morphology

The majority of feces attributed to hyenas were white or pale yellow in color, and spheroidal, oval, or subspherical in shape.

**Figure 2:**
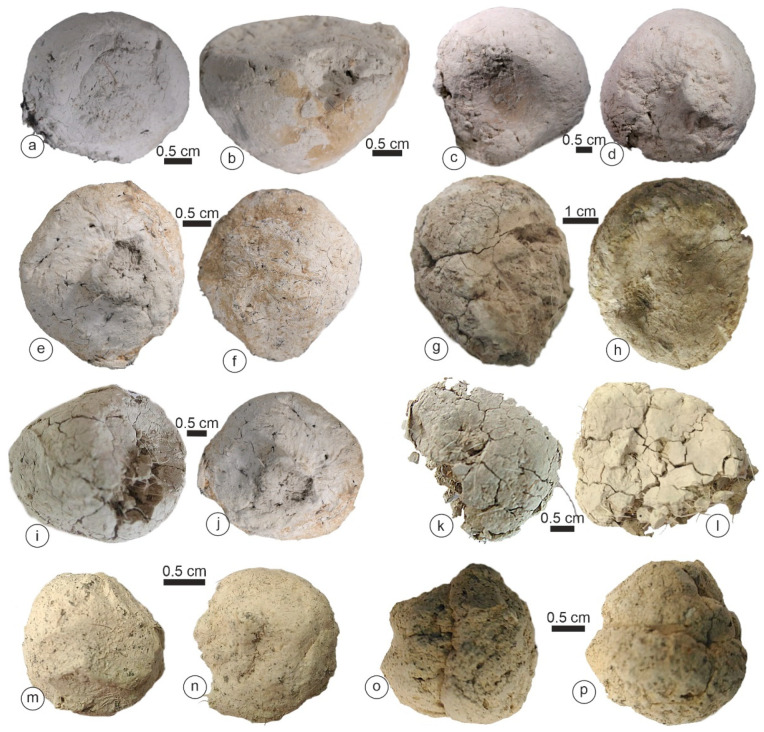
The morphology of the *Crocuta crocuta* samples. (**a**) Tchad. (**b**) Tanzania. (**c**,**d**) Soysambu, Kenya. (**e**,**f**) Samburu/Seva Cons., Kenya. (**g**–**l**) Kipeto, Kajyado, Kenya. (**m**–**p**) Barogali, Djibouti.

*Crocuta* feces were bigger than those of other species, with a maximal size of 38.5 mm (Table 1). They exhibited a more or less triangular “flat” face (Figure 2b,h,i). An outer thick layer (cortex) was sometimes distinguishable (Figure 2a,c,h). Most samples were compact and hard, while others were friable (Figure 2k). Bone or tooth remains were not visible on the outer surface of these samples.

**Figure 3:**
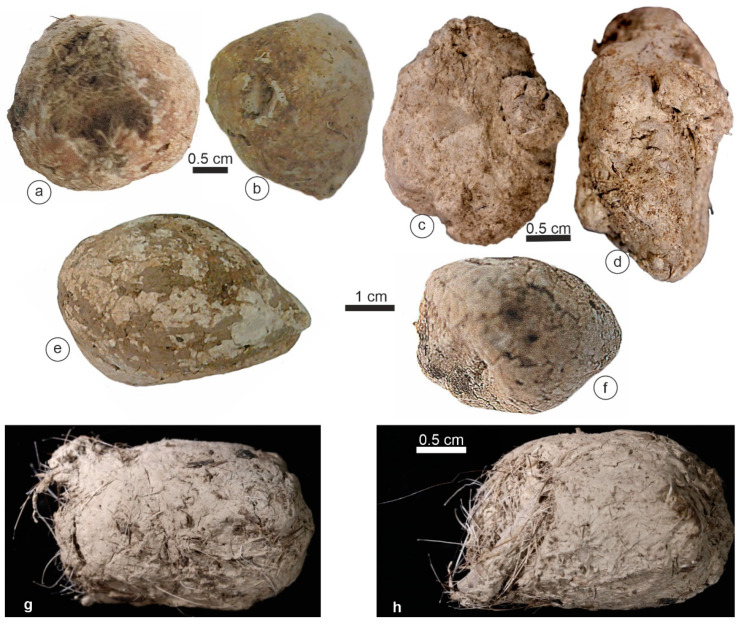
The morphology of the *Hyaena hyaena* samples. (**a**,**b**,**e**,**f**) West Turkana, Kenya. (**c**,**d**,**g**,**h**) Shompole, Kenya.

The morphology and color of *Hyanea* coprolites were similar to those of *Crocuta* (Figure 3). Typically, the outer surface of the samples was hard, and they were friable and exhibited numerous hairs (Figure 3g,h). The maximal size was 28.5 mm (Table 1).

**Figure 4:**
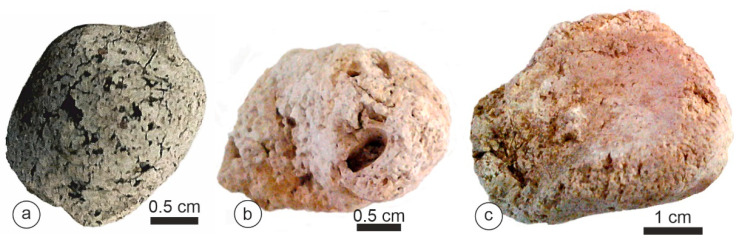
The morphology of the *Parahyaena brunnea* samples. (**a**) Limpopo, BRS, South Africa. (**b**,**c**) Gewihaba Hills, Botswana.

*Parahyaena* coprolites had an irregular outer surface (Figure 4) and reached a maximum size of 20–25 mm (Table 1). Despite their porous appearance, with both small and large holes (Figure 4), these coprolites were remarkably hard.

**Figure 5:**
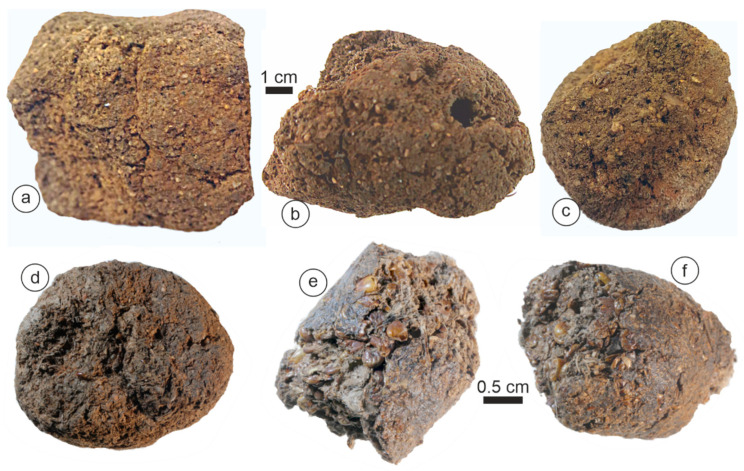
(**a**–**f**) The morphology of the *Proteles cristatus* samples. Samburu, Kenya.

*Proteles* coprolites were characterized by a distinct appearance: brown and tube-shaped. The largest collected samples reached 90 mm (Table 1). These scats exhibited a sandy texture and were highly friable, and we observed the remains of insects, such as termites, on the surface or on the broken sides (Figure 5).

### 3.2. Contents

**Figure 6:**
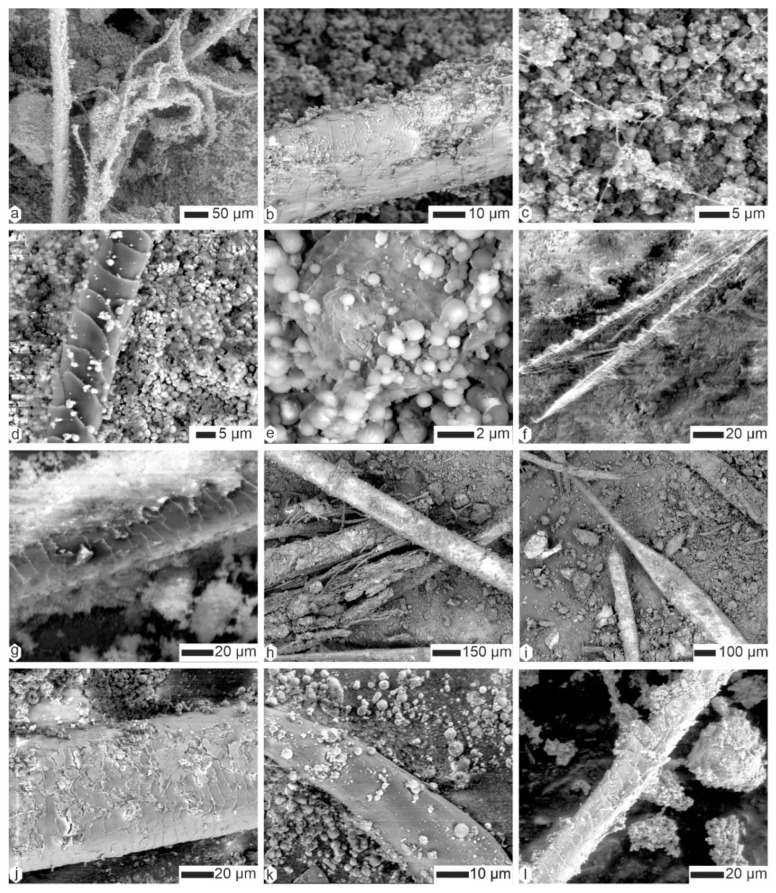
The contents of the superficial layer of coprolites of *Crocuta crocuta*. (**a**,**b**) Filaments and hairs, CC-Tchad. (**c**) Thin filaments (hairs?) and small rounded particles (bacteria?), CC-Tchad. (**d**) Hair and small round particles, CC-Tanz. (**e**) The same sample, showing details of the round particles (bacteria?). (**f**) A longitudinally split plant fiber, CC-soy. (**g**) The same sample, showing a partially embedded hair in the matrix. (**h**,**i**) Hair and plant fibers, CC-sam. (**j**) Altered hair, CC-djib2. (**k**) A plant fiber in the same sample. (**l**) Hair, CC-djib1.

**Figure 7:**
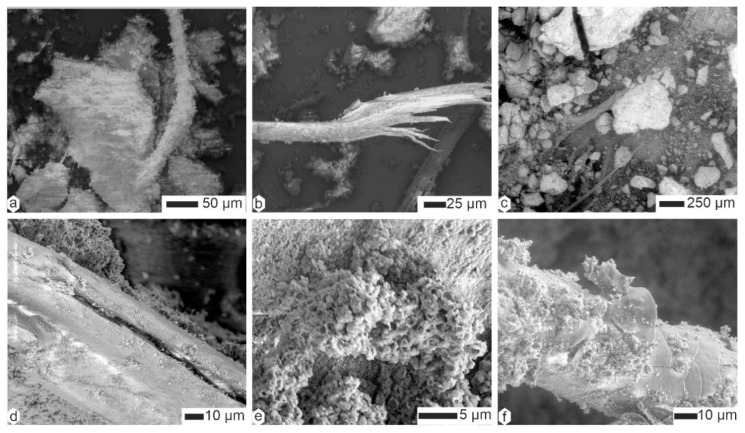
Analysis of the superficial layer of Kipeto (Kajiado, Kenya) *Crocuta crocuta* coprolites reveals plant fibers and hairs embedded within a finely granular matrix. (**a**,**b**) Plant fibers, CC-kip5. (**c**,**d**) Plant fibers from CC-kip4. (**e**) Matrix, CC-kip. (**f**) Hair, CC-kip.

Coprolites of *Crocuta* consisted of organic fibers embedded within a matrix of tiny mineral particles (Figure 6 and Figure 7). The diverse diameters and surface structures of these fibers indicated their varied origins. Some fibers showed a scaly surface, and so were assigned to hairs, whereas others were branched and folded (Figure 6a). Depending on the sample, these fibers were often covered with small round particles, usually assigned to bacteria (Figure 6a,b,d,e). Very thin filaments also existed, the diameter of which being smaller than 1 µm (Figure 6c). Some remains were composed of elongated aligned elements, similar to dry vegetal structures (Figure 6f,i and Figure 7a–d), whereas others definitely had the appearance of hair (Figure 7f).

Samples from Kipeto contained a mixture of plant sprigs and hairs, with the scaly structure of the hairs being more or less preserved (Figure 7). It is currently unclear whether this plant material is digested or originates from the surrounding soil. We can, however, bear in mind that many carnivores start to eat the rumen of their herbivore prey.

**Figure 8:**
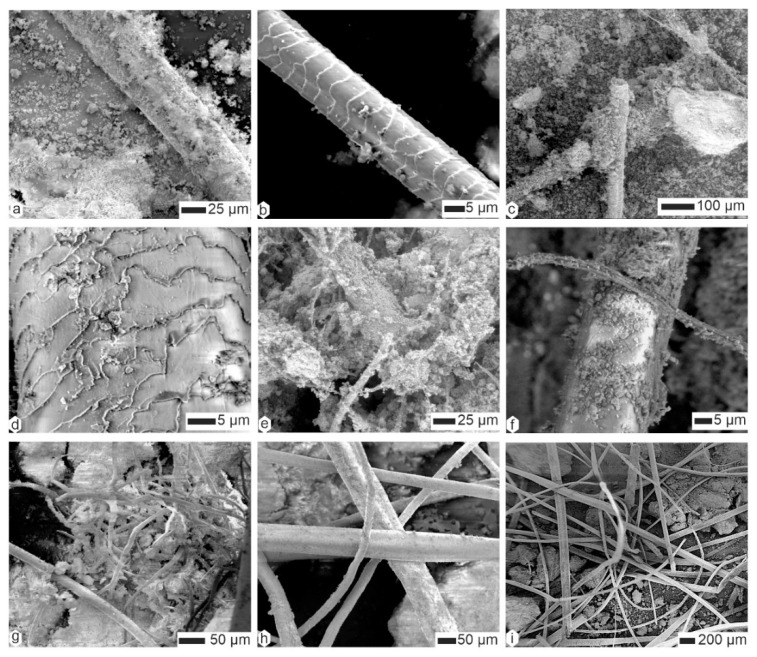
Contents of the superficial layer of coprolites of *Hyaena hyaena*. (**a**,**b**) Scaly hairs, HH-Lom. (**c**,**d**) Hairs and microgranular matrix, HH-M113. (**e**,**f**) Hairs and plant fibers more or less embedded in the matrix, HM-LA2c. (**g**) Fibers and hairs entangled, FF-F105. (**h**,**i**) “Clean” hairs of different diameters, FF-F105.

Hairs were also present in the coprolites of *Hyaena* (Figure 8a–d); they could be taxonomically determined (i.e., morphology of scales, ratio of medulla to cortex) [42,43]). In a given sample, they were often covered with small, round particles, often described as bacteria (Figure 8c–d). When these “bacteria” obscured the surface of the filaments, it became difficult to distinguish between hairs and plant sprigs (Figure 8e,g,h). Felts of fine filaments of unknown origin also occured (Figure 8i). The diameter of the preserved hairs varied.

**Figure 9:**
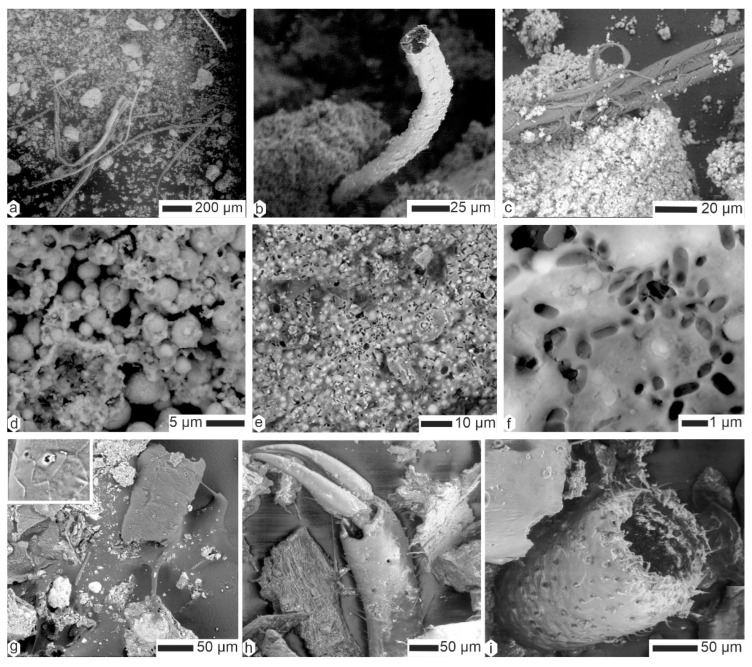
(**a**–**f**) Contents of the superficial layer of coprolites of *Parahyaena brunnea*. (**a**) Hairs and plant fibers. (**b**) Scaly hair and granular matrix, HB-lim. (**c**,**d**) Plant fiber and granular matrix, HB-p**.** (**e**) Round particles of the granular matrix, HPg. (**f**) Oval holes, probably related to bacteria, HBg. (**g**–**i**) Contents of the superficial layer of coprolites of *Proteles cristatus.* (**g**) A small fragment of hair. (**h**,**i**) Remains of insect mandibles and cuticle.

Plant fibers and hairs were also visible in *Parahyaena* coprolites (Figure 9a–c). The matrix was microgranular, as shown in Figure 9c–e. High-magnification images of the matrix revealed numerous microcavities, often described as remnants of dissolved bacteria [44]. These microcavities were approximately 1 µm in length (Figure 9f).

Unlike *Parahyaena* feces, *Proteles* feces contained few, if any, plant fibers or hairs (Figure 9g). However, biological remains were abundant, including insect mandibles (Figure 9h) and other indigestible materials (Figure 9i).

### 3.3. Composition

#### 3.3.1. Global Data

**Figure 10:**
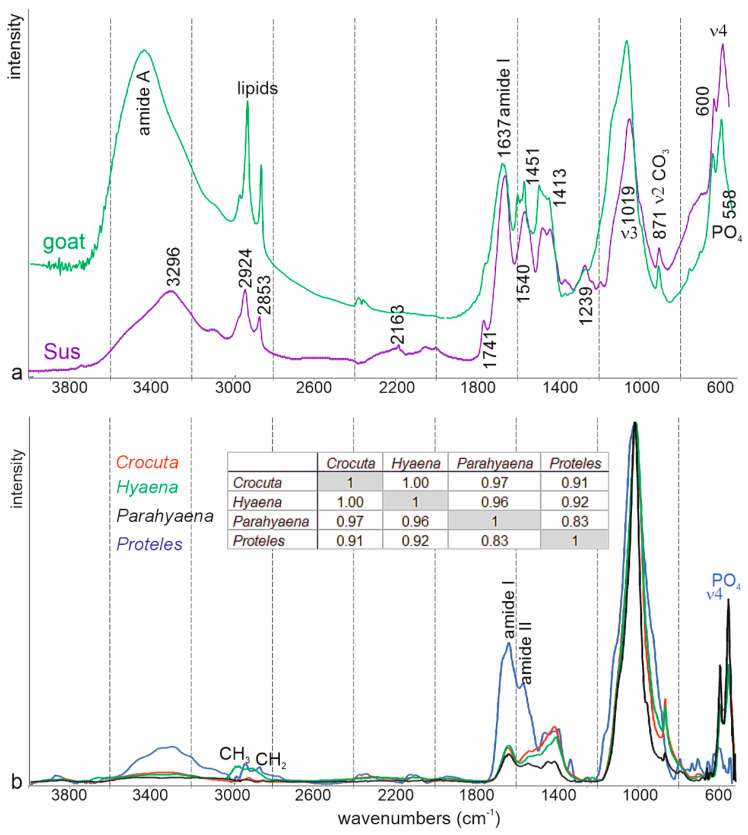
FTIR ATR average spectra of mammal bones (**a**) and *Hyaenid* coprolites (**b**). *Hyaenid* and *Sus* bone spectra were compared to the standards library of the Kimmel Center for Archaeological Science, available online at http://www.weizmann.ac.il/kimmel-arch/infrared-spectra-library (accessed on 11 October 2024). Insert: linear correlation coefficients.

Figure 10a presents two examples of mammal bones. The studied fragments were left un-etched, ensuring the preservation of all components. Although the preparation methods differed (KBr + bone powder pellets for goat vs. ATR FTIR powdered for *Sus* [45]), the key spectral characteristics of both taxa are comparable. Organic bands, indicative of proteins and lipids, exhibit strong intensities. However, potential bands attributed to “sugars” are obscured within the slope of the ν3 PO_4_ band.

The FTIR spectra of the *Crocuta* coprolites differ from those of modern fresh bones (Figure S1, Figure 10). In the coprolite spectra, bands associated with organic components are weak or absent: amide I and II bands are diminished, the amide A band at approximately 3300 cm⁻^1^ is barely noticeable, and amide III bands are not detected. Lipid bands are also absent, except in spectra for samples from Samburu (CC-Sam) and Kipeto (CC-kip5). Bands associated with PO_4_ (~1015 cm⁻^1^) and CO_3_ (~1420 cm⁻^1^) are better preserved. Although bone contains CO_3_, it cannot be concluded that calcite is present as a secondary embedding mineral. In the spectra of fresh *Sus* scrofa bone, the ν4 PO_4_ domain exhibits two bands: one at 559 cm⁻^1^ and a weaker one at 600 cm⁻^1^. This suggests that neither *Sus* bones nor coprolites are enriched with fluoride [46]. In the spectra of these samples, CO_3_ and PO_4_ bands are likely indicative of the presence of apatite.

*Proteles* coprolites are richer in organic components than those of *Hyaena* and *Crocuta* (Figure 10). Amide A, lipids, and amide I, II, and III bands are visible in the spectra of *Proteles* coprolites, while CO_3_ and PO_4_ bands are weak or absent. Additionally, the ν4 PO_4_ doublet (~550–600 cm⁻^1^) is absent, suggesting that phosphate may not be present in the form of apatite.

#### 3.3.2. Statistical Analyses

##### Selection of the “Mineral” Bands

Multiple linear regression analysis of the entire FTIR spectra revealed the highest coefficients between *Crocuta* samples from Tanzania and Soysambu (0.995), and between *Parahyaena* from South Africa and *Crocuta* samples from Kipeto (0.993). The lowest coefficient was observed between *Parahyaena* (small piece) and *Crocuta* from Tanzania (−0.097). Notably, most coefficients exceeded 0.9, with the exception of *Parahyaena* and *Proteles*.

The use of FTIR spectroscopy in bone studies is widely documented [47,48,49,50,51,52]. Furthermore, any residual bone fragments may have been digested. Thus, different spectral ranges related to CO_3_ and PO_4_ were explored:

- The surface between 560 and 600 cm^−1^, indicative of PO_4_ in apatite [53];
- The surface between 860 and 880 cm^−1^, indicative of CO_3_;
- The surface between 1409 and 1425 cm^−1^, indicative of CO_3_;
- The surface between 1030 and 1010 cm^−1^, a crystallinity index (CI) [54];
- The CO_3_/PO_4_ ratio was estimated using bands at 871 and 1017 cm^−1^;
- The CO_3_/PO_4_b ratio was estimated using bands at 1415 and 575 cm^−1^ [55].

**Figure 11:**
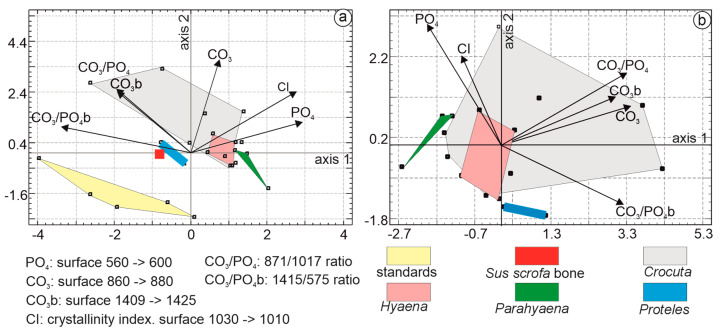
(**a**) Principal component analyses (PCA) based on FTIR mineral bands of coprolites, *Sus* bone, and organic standards. The PCA shows that the first principal axis contains 38.7% of the total variance, while axis 2 represents 32.9%. The first principal component is characterized by higher CO_3_PO_4_b, PO_4_, and Cl eigenvalues. In the second principal component, samples are characterized by higher CO_3_, CO_3_PO_4_, and CO_3_b values. (**b**) PCA based on organic FTIR bands of coprolites. The first principal axis contains 50.2% of the total variance, while axis 2 represents 22.9%. The first principal component is characterized by higher CO_3_, CO_3_PO_4_, and CO_3_PO_4_b eigenvalues. In the second principal component, samples are characterized by higher PO_4_, Cl, and CO_3_b values.

The multivariate analysis employed principal component analysis. The first principal axis accounted for 38.7% of the total variance, followed by axis 2 at 32.94% and axis 3 at 11.64%. The first principal component was characterized by higher CO_3_PO_4_b, PO_4_, and Cl eigenvalues. In the second principal component, samples were characterized by higher CO_3_, CO_3_PO_4_, and CO_3_b values. Organic standards, *Sus* bone, *Proteles*, and *Parahyaena* were clearly differentiated (Figure 11a). The *Hyaena* domain was encompassed within the *Crocuta* domain.

To better visualize the relationships between the coprolites, the analysis was repeated, excluding *Sus* bone and organic standards (Figure 11b). The first three principal axes explained 87.21% of the total variance, with axis 1 accounting for 50.2%, axis 2 for 22.93%, and axis 3 for 14.08%. Axis 1 was characterized by higher eigenvalues for CO_3_, CO_3_PO_4_, and CO_3_PO_4_b. Axis 2 differentiated samples based on higher PO_4_, Cl, and CO_3_b values. The *Crocuta* group formed the largest domain, encompassing *Hyaena* samples. *Proteles* and *Parahyaena* were clearly distinct.

Multiple linear regression analysis was used to identify the strongest correlations among the variables: CI and CO_3_ (0.674), CI and PO_4_ (0.559), and CO_3_B and CO_3_PO_4_b (0.501). Considering the number of samples, their composition, and the correlations between the variables, only three variables were selected: CO_3_, PO_4_, and CO_3_PO_4_b.

##### Selection of the “Organic” Bands

A similar approach focused on organic matrices, using examples such as chitin, keratin, and specific sulfur groups within the 1030–1160 cm^−1^ range [56,57,58]. Cholesterol was represented by the spectral region between 2850 and 2935 cm^−1^. Amide II and I contents were attributed to the regions 1535–1556 cm^−1^ and 1631–1650 cm^−1^, respectively. An overlap was observed between the bands from 960 to 1230 cm^−1^ and those from 930 to 1230 cm^−1^, associated with polysaccharides, keratin, and sulfur groups.

**Figure 12:**
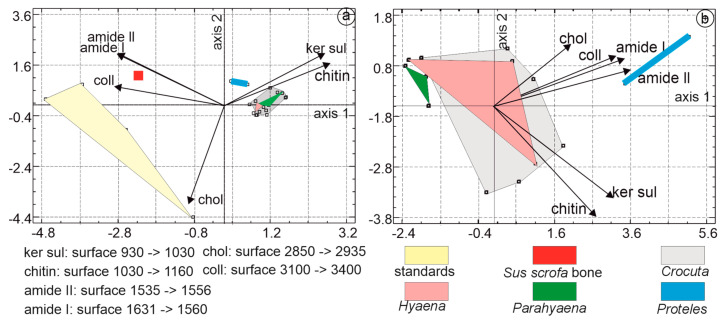
(**a**) Principal component analyses (PCA) based on organic FTIR bands of coprolites, *Sus* bone, and organic standards. The first principal axis accounts for 64.5% of the total variance, followed by axis 2 at 19%. The first principal component is characterized by higher eigenvalues associated with collagen, amide I, and amide II. (**b**) The PCA based on coprolites only shows that the first principal axis contains 62.6% of the total variance, while axis 2 represents 17.5%. The first principal component is characterized by higher eigenvalues associated with amide I, collagen, and amide II. In the second principal component, samples are characterized by higher levels of cholesterol, collagen, and amide II.

In the PCA dedicated to organic components, the first principal axis accounted for 64.5% of the total variance, followed by axis 2 at 19% and axis 3 at 12.2%. The first principal component was characterized by higher eigenvalues associated with collagen, amide I, and amide II. The second principal component differentiated samples based on higher levels of cholesterol, keratin sulfate, and amide I. Organic standards, such as *Sus* bone and *Proteles*, were readily identifiable. Notably, *Sus* bone was positioned close to collagen. The domains of *Hyaena*, *Crocuta*, and *Parahyaena* overlapped (Figure 12a).

To enhance the visualization of the relationships between samples, the same analysis was conducted, excluding *Sus* bone and organic standards. The first principal axis accounted for 62.64% of the total variance, followed by axis 2 at 17.5% and axis 3 at 13.01%. The first principal component was characterized by higher eigenvalues associated with amide I, collagen, and amide II. The second principal component differentiated samples based on higher levels of cholesterol, collagen, and amide II. The largest domain belonged to *Crocuta*, and partially overlapped with the domain of *Hyaena*. *Proteles* and *Parahyaena* were clearly distinct (Figure 12b).

Multiple linear regression analysis was employed to identify the strongest correlations among the organic variables: this was found to be between amide I and amide II, revealing a high correlation of 0.99. Considering the number of samples, their composition, and the intercorrelations between variables, a parsimonious model was selected, including only three variables: chitin, cholesterol, and amide I.

##### “Mineral” and “Organic” Bands

PCA was performed using the organic and mineral bands selected in previous steps: CO_3_, PO_4_, and CO_3_PO_4_b for mineral components, and chitin, cholesterol, and amide I for organic components.

**Figure 13:**
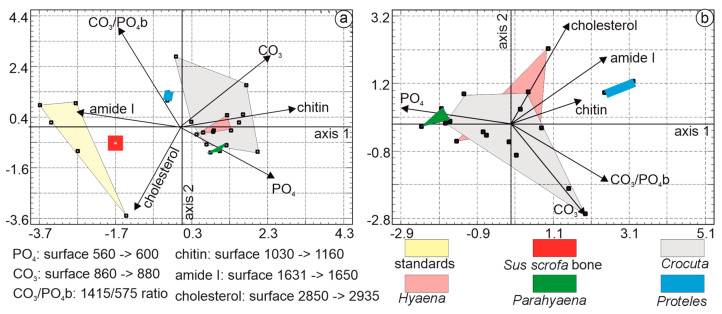
(**a**) Axis 1 explains 48.20% of the total variance, followed by axis 2 at 24.24%. The first principal component is characterized by high chitin, amide I, and PO_4_. The second principal component differentiates samples based on levels of CO_3_, amide I, and PO_4_. (**b**) The first principal axis explains 39.7% of the total variance, and axis 2 explains 21.4%. Axis 1 is primarily characterized by higher eigenvalues associated with PO_4_, CO_3_/PO_4_b, and CO_3_, while axis 2 differentiates samples based on levels of cholesterol, amide I, and CO_3_.

The first principal axis of PCA, based on coprolites, *Sus* bone, and organic standards accounted for 48.20% of the total variance, followed by axis 2 at 24.24% and axis 3 at 13.71%. The first principal component was characterized by higher eigenvalues associated with chitin, amide I, and PO_4_. The second principal component differentiated samples based on higher levels of CO_3_, amide I, and PO_4_. Organic standards, *Sus* bone, and *Proteles* were readily identifiable (Figure 13a). The untreated bone fragment was rich in organic matrix. The domain of *Hyaena* and a small region of *Parahyaena* fell within the domain of *Crocuta*.

In the PCA dedicated to the organic components of coprolites (Figure 13b), the first three principal axes explained 79.2% of the total variance, with axis 1 accounting for 39.7%, axis 2 for 21.4%, and axis 3 for 18.1%. Axis 1 was primarily characterized by higher eigenvalues associated with PO_4_, CO_3_/PO_4_b, and CO_3_, axis 2 differentiated samples based on higher levels of cholesterol, amide I, and CO_3_.

Samples from *Hyaena*, rich in cholesterol, exhibited some overlap with the *Crocuta* domain. Overall, *Hyaena* samples were richer in organic components, while *Crocuta* samples were richer in minerals, particularly CO_3_. *Parahyaena* samples clustered closely with those of *Crocuta*. The absence of carbonates and phosphates and the presence of organic components (mainly chitin) in the diet of *Proteles* were evident.

## 4. Discussion

### 4.1. Collecting and Extracting Coprolite Samples

All samples were collected from their natural environments to eliminate biases associated with the standardized diets provided to animals in zoos. The size and location of the samples also presented constraints. Coprolites are not uncommon in nature, so invasive analyses remain possible. One of our primary objectives is to develop methods capable of extracting data from the smallest possible quantities of material. We are initially focusing on recent samples, before we apply these methods to archeological and fossil coprolites.

Two main categories of analyses and observations are available: taking a localized sample, or taking a sample without a location. Most coprolites are dry and hard. To best preserve their physical integrity, the sample to be analyzed was extracted from the outer surface of the coprolite, and was approximately 5 mm deep or less than 5 g.

### 4.2. Observing and Analyzing Coprolite Samples

Typically, only the shape, size, and weight of coprolites are described and considered for predator identification. In our study, the predator was known, but the challenge lay in determining the diet from a microsample with statistical significance, while preserving the integrity of the entire specimen.

Fourier transform infrared spectrometry (FTIR) has been extensively used to analyze both healthy and pathological bone. Using the attenuated total reflectance (ATR) mode, samples weighing less than 1 g are sufficient to obtain valuable spectra. Moreover, neither inclusion nor polishing procedures are required, minimizing contamination. Notably, acquiring spectra is a rapid and inexpensive method. Importantly, the same sample can be examined using scanning electron microscopy (SEM) and analyzed using energy-dispersive X-ray spectroscopy (EDS).

### 4.3. Comparison with Other Methods

Coprolites are used to identify both the remains they contain, which may be prey for carnivorous animals, and the organisms that produced them, such as predators. Shape and size are the most commonly used parameters to identify the predator. Although internal structures and contents are sometimes described, they are primarily descriptive, including bone fragments, insects, and pollen. Since the studies of Hausman [59,60], characteristics of the scaly surface of hairs and the shape of sections (round, elliptical, etc.) have been used to identify isolated samples [61]. While these analyses are effective in identifying both prey and predator, they are unfortunately destructive. To determine the prey, the coprolite must be dissected.

### 4.4. Cross-Validations of the Analyses

In this study, dried coprolite samples were collected from the natural environments of both predators and prey. These samples were found either in isolation, or dispersed in soil, with or without grass. Given their locations, we anticipated the presence of argillaceous and silt particles on the extracted samples. SEM images confirmed the presence of grass fragments and sedimentary particles, but FTIR spectra did not reveal silicates or aluminosilicates. EDS analyses showed significant variability in the chemical element contents of each sample, particularly in terms of phosphorus, calcium, and silicon.

FTIR spectra of clay and aluminosilicate particles exhibit characteristic bands in the 1000–1030 cm^−1^ and 1400–1600 cm^−1^ regions, which overlap with those attributed to PO_4_ in coprolites. This suggests that some of the PO_4_ bands may have been composite features arising from both bone and soil. However, the diverse geographical and environmental origins of our modern samples make it unlikely that the “common” bands are solely attributable to sediment. Therefore, a combination of bone and soil components is more probable.

## 5. Conclusions

Analyzing coprolites offers the most reliable method for identifying a producer, though this can be challenging. The remains found within coprolites are fragmented and usually small, hindering identification. Furthermore, even coprolites from carnivorous animals may contain traces of grass and pollen. While hairs are often present, they are usually unidentifiable, due to the limited catalog of hair samples, which currently only covers some large animals. Another common type of remain is often described as bacteria. These rounded corpuscles are consistent with bacteria in size and shape; however, their internal structure and composition remain unknown.

This study analyzed the overall composition of the coprolite matrix within a single family: *Hyaenidae*. Scanning electron microscope (SEM) observations revealed an absence of identifiable bone or tooth fragments in the superficial layers of the coprolites, despite the abundance of hair. While bone microfragments were not particularly abundant in our samples, their presence within coprolites is a common observation in other analyses [3]. The abundance of plant remains alongside the absence of pollen presents a paradox, especially considering the broad geographical origin of the samples and the typical resilience of sporopollenin. To better understand and to detect digestive alterations, we incorporated organic standards known to be present in bone, as well as a modern bone sample. Fourier transform infrared attenuated total reflectance (FTIR-ATR) spectroscopy, a simple, inexpensive, rapid, and non-destructive method, was used for sample analysis, notably allowing for quantification.

Classical analyses of fossil bones and teeth rely on morphology and chemical composition. While predation is assumed to be the primary cause of death, the processes occurring between the time of death and fossilization are frequently overlooked. Specifically, the effects of digestion, a significant factor influencing the preservation of bones and teeth, are often neglected. This oversight can introduce biases into reconstructions of ancient environments.

Our method bypasses the need for identifying fragmentary remains, instead utilizing the powdered coprolite matrix. Preliminary results, derived from image analysis and statistical evaluation of infrared spectrometry data, show promise. However, further validation with a larger sample set is required.

## Figures and Tables

**Table 1:** The species, origin abbreviations, and size of the studied samples.

Species	Country	Region	Collector/Date	# This Study	L x ep (mm)	Note
*Crocuta crocuta*	Kenya	Kipeto, Kajiado	J.P. Brugal, 2019	CC-kip4	28x27.5	isolated
	Kenya	Kipeto, Kajiado	J.P. Brugal, 2019	CC-kip5	28x38.5	isolated
	Kenya	Kipeto, Kajiado	J.P. Brugal, 2019	CC-kip	-	isolated
	Kenya	Samburu/Seva Cons.	T. Adhola, 2017	CC-Sam	38x27	isolated
	Kenya	Soysambu	J.P. Brugal, 2017	CC-soy	34x28	isolated
	Djibouti	Barogali	J.B. Fourvel, 2019	CC-djib1	24x24	isolated
	Djibouti	Barogali	J.B. Fourvel, 2019	CC-djib2	-x19.5	isolated
	Tchad	Zakuma, Bar Salamat	C. Denys, 2000	CC-Tchad	27x25	isolated
	Tanzanie	Kingu Pira	C. Denys, 2003	CC-Tanz	32x21	isolated
*Hyaena hyaena*	Kenya	Shompole	O. Mwebi, 2009	FF-F105	19x9.5	isolated
	Kenya	Shompole	O. Mwebi, 2008	HH-M113	24x16	isolated
	Kenya	West Turkana	J.P. Brugal, 2012	HM-LA2c	28.5x22	isolated
	Kenya	West Turkana	J.P. Brugal, 2013	HH-Lom	23x18.5	isolated
*Parahyaena brunnea*	South Africa	Limpopo, BRS	J.P. Brugal, 2016	HB-lim	20x15.7	isolated
	Botswana	Gewihaba Hills	J.B. Fourvel, 2021	HBp	small specimen	isolated
	Botswana	Gewihaba Hills	J.B. Fourvel, 2021	HBg	larger specimen	isolated
*Proteles cristatus*	Kenya	Samburu	J.P. Brugal, O. Mwebi, 2017	Pro	90x85	latrine
	Kenya	Samburu	J.P. Brugal, O. Mwebi, 2017	Pro2023	30x18	latrine

## Data Availability

The underlying analytical data are still being used for research, and are therefore proprietary.

## Supplementary Materials

The following supporting information can be downloaded at: https://www.mdpi.com/article/10.3390/ani15081145/s1, Figure S1: FTIR spectra of organic and mineral standards.
