# Supplementary figures and images for: Coprolite Multimodal Analysis: A Tool for Hyaenid Predator Identification
Source: Animals (Basel). 2025 Apr 16;15(8):1145. doi: 10.3390/ani15081145 (PMC12024366; doi:10.3390/ani15081145)

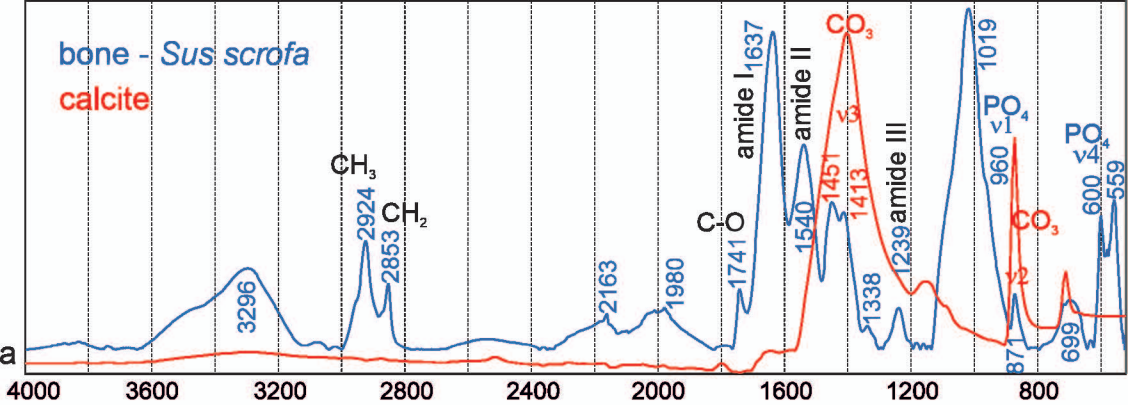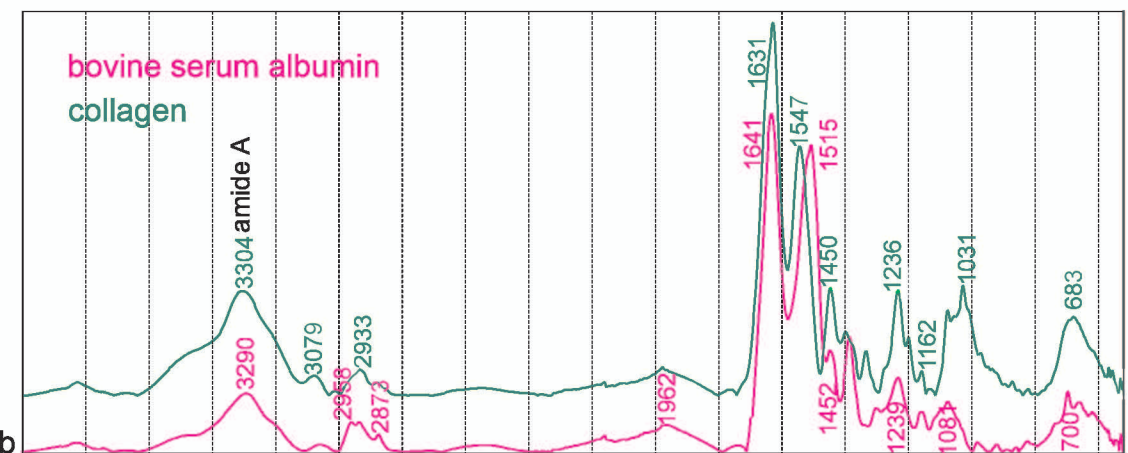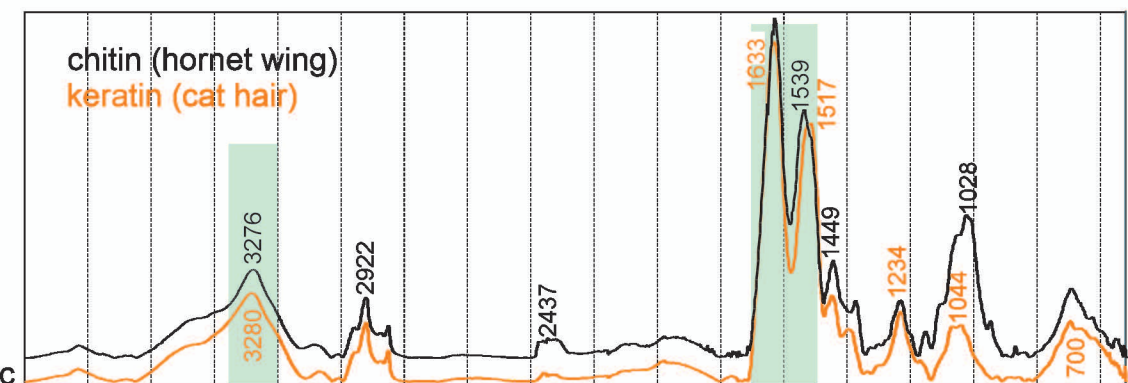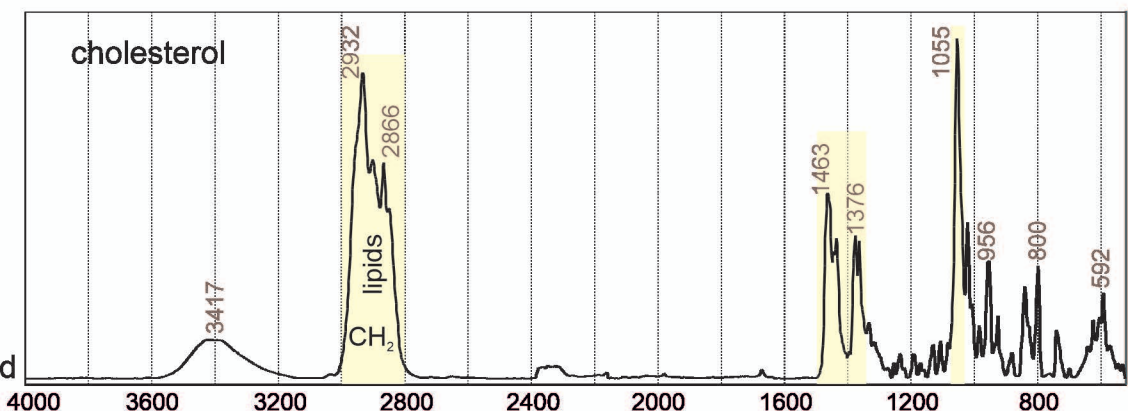

Supplement: Supplementary file 1 [file animals-15-01145-s001.zip › animals-3545879-supplementary.pdf]
